# Supplementary figures and images for: Neutralization of p40 Homodimer and p40 Monomer Leads to Tumor Regression in Patient-Derived Xenograft Mice with Pancreatic Cancer
Source: Cancers (Basel). 2023 Dec 11;15(24):5796. doi: 10.3390/cancers15245796 (PMC10742282; doi:10.3390/cancers15245796)

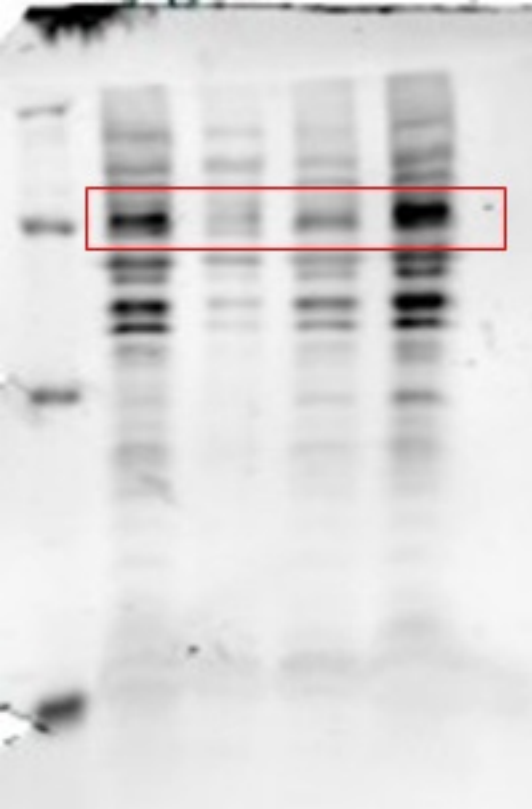

Bcl2

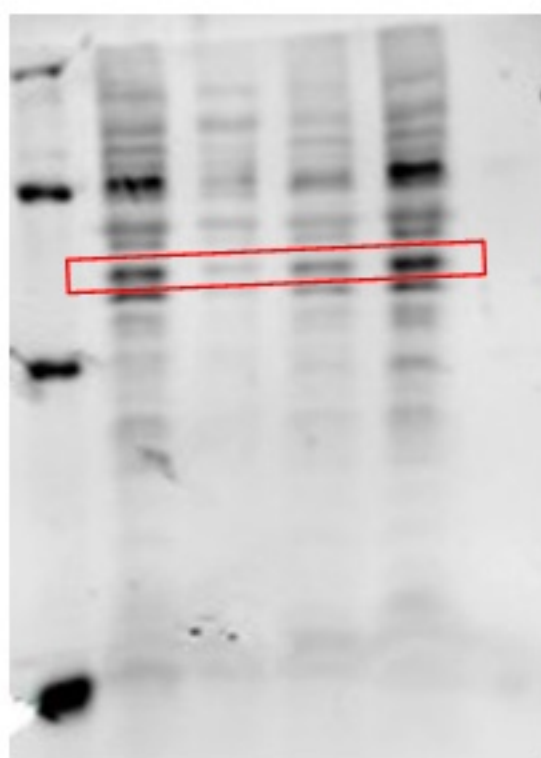

p-Bad

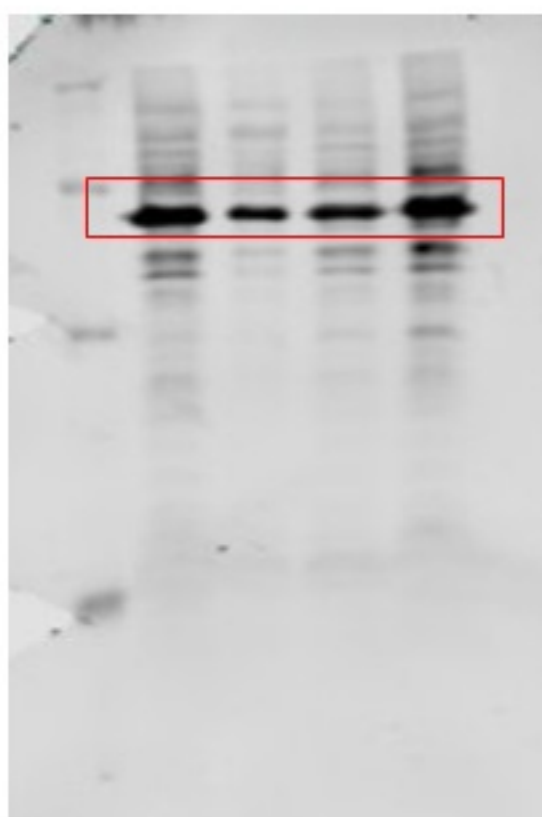

$\beta$ -actin

Supplemental Figure S1 Raw Western blots for Figure 8G.

Supplement: Supplementary file 1 [file cancers-15-05796-s001.zip › cancers-2695967-supplementary.pdf]
